# The Genius of the Zebrafish Model: Insights on Development and Disease

**DOI:** 10.3390/biomedicines9050577

**Published:** 2021-05-20

**Authors:** James A. Marrs, Swapnalee Sarmah

**Affiliations:** Department of Biology, Indiana University—Purdue University Indianapolis, 723 West Michigan, Indianapolis, IN 46202, USA; ssarmah@iu.edu

The zebrafish is an outstanding and inexpensive vertebrate model system for biomedical research. The embryos develop externally allowing experimental access treatments; are transparent allowing for microscopy visualization; develop rapidly; and have outstanding genetic and genomic resources. The zebrafish model was first developed to study neural development, and this tradition continues. A large-scale mutagenesis screen propelled the zebrafish model rapidly forward to become the modern research platform we see today. Creative approaches using the zebrafish were developed to study toxicology, disease etiology, behavioral neuroscience and regenerative biology. Using these experimental foundations, the contributions found in this Special Issue of Biomedicines illustrate the power and potential of the zebrafish.

Due to evolutionary conservation among vertebrate organisms, animal models provide insight into disease processes that cannot be obtained by studying human patients. The genetic conservation between vertebrates allows scientists to evaluate consequences of mutations in disease etiology. There is an art to developing a useful disease model for biomedical research, and the zebrafish are being used at an increasing frequency to evaluate disease development and consequences. The genetic resources are being developed in the zebrafish, and it has been an established model system to determine the etiology of genetic disorders caused by damaging mutations in protein-coding genes. The result of the Human Genome Project led to the recognition of single-nucleotide polymorphisms (SNPs) that increase or decrease the risk for a given disease. Patients’ genomic analyses have identified many clinically relevant mutations in the noncoding regions of the human genome which likely disrupt functions of cis-regulatory elements (CREs). A review by Mann and Bhatia [1] highlights the utility of the zebrafish for studying mutations in noncoding regions of the genome. The zebrafish offer low maintenance cost, short generation times, imaging approaches and transgenic technology which can be exploited to evaluate functional consequences of noncoding region mutations.

A couple of reviews discuss how the zebrafish are used to study heart disease models. Giardoglou and Beis [2] discuss the utility of the zebrafish in examining the heart and vasculature. The zebrafish heart shares features with the human heart not found in other models, and consequently, disease models share similarities in pathophysiological consequences with the human conditions. They also highlight the use of genome-wide association studies (commonly referred to as GWAS) to identify genetic variants (multiple loci and SNPs associated with coronary artery disease and cardiomyopathies) and the use of the zebrafish to dissect functional defects and validate candidate causal genes that emerge from GWAS analyses. Santoso et al. [3] review the methodology used to study cardiac rhythm in the zebrafish. There are experimental challenges associated with a small aquatic organism, but methods are continually improving to detect cardiac rhythm in both embryonic and adult zebrafish. The authors discuss current methodologies including the dynamic pixel change method, kymography, laser confocal microscopy, artificial intelligence and electrocardiography (ECG). The authors also provide perspectives on advances like imaging technology and artificial intelligence that are applied to studies of cardiac rhythm.

Parkinson’s disease genes and effects of environmental toxins have been uncovered in recent years. Barnhill et al. [4] review the use of the zebrafish as a model of Parkinson’s disease. Individual genes and the zebrafish models affecting the progression of a Parkinson’s disease phenotype are discussed. These genes point to protein aggregation, protein degradation, mitochondrial dysfunction, synapse dysfunction, inflammation and cell death pathways. Environmental assaults are also studied in the zebrafish, which fit into similar pathophysiological pathways as the genetic defects.

Two research articles provide fundamental insight into biological processes that affect muscle and eye conditions. Fallata et al. [5] examined gelatinase A (a matrix metalloproteinase) expression and activity in muscles, which participates in myosin degradation. They showed that gelatinase A has poorly recognized signal sequences and conserved phosphorylation sites, which may regulate its enzymatic activity within the sarcomere. The gelatinase A enzyme has not been characterized in specific diseases, but the authors discussed the physiological role, and the evidence suggests potential roles in disease and injury.

An exciting study by Brastrom et al. [6] produced a model of microphthalmia (small eye phenotype) caused by mutations in the human RBM24 gene, an RNA-binding protein. This RNA-binding protein regulates the gene most commonly affected in microphthalmia patients, SOX2. The experiments showed that overexpression of the zebrafish Sox2 protein suppresses the microphthalmia phenotype, supporting the posttranscriptional role for the Rbm24 protein in binding and targeting the *sox2* mRNA.

Neuroscience of the zebrafish is at the foundation of this model organism due to the transparency of embryos allowing neural development visualization. Neuroscience functional changes can be studied using animal behavior analysis. Since fish have very different lives from humans, the analysis of fish behavior is challenging. Progress is being made with the study of zebrafish behavior and correlating fish behavior with that of humans. Zebrafish behavior is being correlated with neural development, electrophysiology and other aspects of neurobiology. A series of articles in this Special Issue of Biomedicines address neuroscience and behavior.

A review article by Basnet et al. [7] discussed the uses of the zebrafish model to evaluate and screen neuroactive drugs. They highlight the studies of the brain–behavior connection and the types of behavior assays that can be used with the zebrafish. Finally, they review studies of neuroactive drugs and toxicants that produce behavior defects in the zebrafish. The review is an excellent primer for the zebrafish behavior model, which is a growing model with outstanding potential.

Using adult zebrafish, Chung et al. [8] use a pentylenetetrazol (PTZ)-induced epileptic seizure model to evaluate anti-convulsive activities of a protein extract from a medicinal plant, *Orthosiphon stamineus*. Intraperitoneal injection of the protein extract decreased seizures and altered neurotransmitter levels in zebrafish brains. Proteomic analysis of zebrafish brains showed differential expression of proteins, particularly a regulator of the trans-SNARE complex that affects neurotransmitter exocytosis. The findings suggest potential mechanisms of antiepileptic drug action that could be further studied.

Two articles describe the development of methodology to evaluate visual responses using the zebrafish. Color preference is a useful behavioral parameter, but Siregar et al. [9] note that there are some apparent contradictions in the literature using this assay. Thus, they test several variables (light source position, light intensity, gender, age, strain, etc.) in order to standardize the method. This is a very useful study that will help the zebrafish community generate results from different laboratories that can be compared to one another. We expect that such standardization and harmonization of methodology will be completed for many assays as the zebrafish model grows.

Optomotor response is a common assay used for animal behavior, and it is an important assay used for the zebrafish. Branstrom et al. [10] produced a simple optomotor assay platform that can be performed with a tablet computer and a cell phone camera. The authors validate the assay using mutant and morpholino knockdown zebrafish larvae that disrupt the visual system. This clever and reproducible assay can be set up without expensive equipment and performed at relatively high throughput.

Regenerative medicine research grows because of the potential to treat defects and diseases that are intractable to current medical technology. The potential of these therapies is huge, but one must temper this excitement with the realization that basic research is needed to learn how the powerful potential can be controlled and applied.

Massoz et al. [11] reviewed the regenerative potential of the zebrafish and its use in the study of potential disease treatments. They focused on important targets for regenerative medicine, heart, liver, pancreas, spinal cord, brain and retina, which are the subject of numerous zebrafish studies. The authors note that it may be a long, difficult path to produce regenerative medicine therapeutics, but basic research using the zebrafish will help facilitate this journey to producing efficacious and safe treatments.

Our laboratory, in collaboration with colleagues from Lilly Research Laboratories, examined [12] the effects of a small-molecule Wnt signaling pathway activator on zebrafish caudal fin regeneration. Fin rays are bony structures, and fin regeneration is a useful model for bone growth and repair. Our studies illustrate that zebrafish fin regeneration is a potent platform to study bone healing and characterize drugs that stimulate regenerative potential.

Recreational drug use and abuse is a public health problem, and exposure to drugs during pregnancy is growing due to the ongoing opioid crisis and marijuana legalization. In a review from our laboratory and colleagues from Brazil [13], experiments using the zebrafish to model effects of marijuana and opioid exposure during development were discussed. The effects of exposure to these drugs during pregnancy are less severe than ethanol exposure and are poorly understood. However, growing evidence indicates that there are lasting defects caused by marijuana and opioid exposure. Our review discusses the zebrafish endocannabinoid and opioid signaling system in the zebrafish and the use of this model for understanding the drug exposure-associated developmental defects and identifying treatments.

Amin et al. [14] examined the effects of embryo exposure to the intoxicating ingredient in marijuana, THC, on the nervous system and muscle development. THC exposure during early development affected the Mauther cell, a large neuron that helps control the escape response in the zebrafish and related organisms. THC-exposed zebrafish also exhibited changes in muscle cells. Swimming behavior was affected by THC exposure, showing that there are significant consequences of THC exposure during development that are evident in the zebrafish model.

An impressive and diverse set of articles are found in the Special Issue of Biomedicines “Zebrafish Models for Development and Disease” that represent a significant scope of the zebrafish research arena. The common themes of the Special Issue include neuroscience, behavioral analysis, disease models and regenerative biology. These topics bridge the foundations of the zebrafish model with the areas that are growing in the field, like behavior and regeneration. The zebrafish model still has potential to spare. The model is used to test ecological toxicology, evolutionary biology, cancer biology and many other topics. There is still untapped potential of the zebrafish model that will be exploited in the future. Efforts to use the zebrafish to screen for new therapeutics are ongoing, but it seems clear that there is still room for growth. The CRISPR/Cas9 gene editing technology makes new powerful experimental models. For example, humanizing genes in the zebrafish for use in drug discovery is now a real possibility. Certainly, there are limits to the zebrafish model, but as Albert Einstein said, “Everybody is a genius. But if you judge a fish by its ability to climb a tree, it will live its whole life believing that it is stupid.” We can look forward to more genius coming from the zebrafish model!

## Data Availability

Not applicable.
